# Downlisting and recovery of species assessed by the IUCN

**DOI:** 10.1111/cobi.70103

**Published:** 2025-07-10

**Authors:** Mu‐Ming Lin, Richard A. Fuller, In‐Ki Kwon, Kisup Lee, Simba Chan, Wangwang Qiu, Yat‐Tung Yu, Chi‐Yeung Choi

**Affiliations:** ^1^ School of the Environment The University of Queensland Brisbane Queensland Australia; ^2^ School of Environmental Science and Engineering Southern University of Science and Technology Shenzhen China; ^3^ Environmental Research Center Duke Kunshan University Kunshan China; ^4^ Waterbird Network Korea Seoul South Korea; ^5^ Black‐Faced Spoonbill Eco Center Incheon South Korea; ^6^ Japan Bird Research Association Kunitachi Japan; ^7^ Wild Bird Society of Japan Shinagawa Japan; ^8^ The Hong Kong Bird Watching Society Kowloon Hong Kong; ^9^ Division of Natural and Applied Sciences Duke Kunshan University Kunshan China

**Keywords:** conservation funding, conservation prioritization, conservation success, endangered species, flagship species, green status of species, Red List status change, stakeholder engagement, 紅皮書等級變化, 保育優先次序, 保育資金, 物種綠色狀態評估, 旗艦物種, 瀕危物種, 保育成功, 利害關係人參與, espátula menor, especie bandera, especie en peligro, estado verde de la especie, éxito de la conservación, priorización de la conservación, participación de los actores

## Abstract

Despite the increasing number of species assessed for extinction risk by the International Union for Conservation of Nature (IUCN) (163,040 species as of 2024), only about 1 in 1,000 have been downlisted due to genuine population improvement. Although this rare conservation achievement has been widely celebrated in several recent cases, some other downlisting decisions have met with controversy. A primary role of the IUCN is to assess extinction risk. In this role, it must maintain its independence and not be influenced by the public outcry that may occur when a high‐profile species is downlisted, even if well‐established conservation programs may be disrupted or abandoned as a result. We explored the potential positive and negative consequences of downlisting for conservation efforts through case studies of the giant panda (*Ailuropoda melanoleuca*), red‐crowned crane (*Grus japonensis*), saiga antelope (*Saiga tatarica*), and black‐faced spoonbill (*Platalea minor*), which has recently been proposed for downlisting. Although downlisting can enable more effective use of limited resources, these cases highlight potential risks, including weakened legal backing, diversion of resources away from the species, and declining public and political support. The relatively unquestioned downlisting of the saiga antelope illustrates how early and inclusive engagement of local experts, assessors, donors, and other stakeholders can help ensure that decisions are effectively communicated and implemented without jeopardizing species recovery. The IUCN Green Status of Species assessment is a complementary tool to the IUCN Red List and offers a useful measure of conservation progress, which can help decision makers ensure that downlisting does not undermine long‐term conservation efforts.

## Abstract (es)

Recuperación y cambios de categoría de las especies evaluadas por la UICN

**Resumen:**

A pesar del creciente número de especies evaluadas en riesgo de extinción por la Unión Internacional para la Conservación de la Naturaleza (UICN) (163, 040 especies en 2024), sólo 1 de cada 1,000 ha sido retirada de la lista debido a una auténtica mejora de la población. Si bien este raro logro de la conservación ha sido ampliamente celebrado en varios casos recientes, otras decisiones de transferencia a un apéndice de protección menor han causado controversia. Una de las principales funciones de la UICN es evaluar el riesgo de extinción. En este papel, debe mantener su independencia y no dejarse influir por las protestas públicas que pueden producirse cuando se baja de la lista a una especie de gran notoriedad, aunque como consecuencia de ello puedan interrumpirse o abandonarse programas de conservación bien establecidos. Exploramos las posibles consecuencias positivas y negativas del cambio de categoría para los esfuerzos de conservación mediante estudios de casos del panda gigante (*Ailuropoda melanoleuca*), la grulla coronirroja (*Grus japonensis*), el antílope saiga (*Saiga tatarica*) y la espátula menor (*Platalea minor*), cuyo cambio de categoría se ha propuesto recientemente. Aunque el cambio de categoría puede permitir un uso más eficaz de unos recursos limitados, estos casos resaltan los riesgos potenciales, como el debilitamiento del respaldo jurídico, el desvío de recursos de la especie y la disminución del apoyo público y político. El cambio relativamente incuestionado del antílope saiga ilustra cómo la participación temprana e inclusiva de expertos locales, evaluadores, donantes y otras partes interesadas puede ayudar a garantizar que las decisiones se comuniquen y apliquen eficazmente sin poner en peligro la recuperación de las especies. La evaluación del Estado Verde de las Especies de la UICN es una herramienta complementaria de la Lista Roja de la UICN y ofrece una medida útil del progreso de la conservación, que puede ayudar a los responsables de la toma de decisiones a garantizar que la transferencia a un apéndice de protección menor no socave los esfuerzos de conservación a largo plazo.

## IUCN紅皮書降級與物種復原

截至2024年, 受國際自然保育聯盟(IUCN)評估的物種已達163,040種, 但由於族群量上升而獲得降級的僅約千分之一。這類罕見的保育成就近年來屢獲肯定, 然而部分降級決策仍引發爭議。IUCN的核心職責是客觀地評估物種滅絕風險, 即使降級可能導致既有保育計畫受阻或終止, 仍必須維持其獨立性, 不受公眾輿論左右。本研究以大熊貓(*Ailuropoda melanoleuca*)、丹頂鶴(*Grus japonensis*)、高鼻羚羊(*Saiga tatarica*)與近期被提議降級的黑面琵鷺(*Platalea minor*)為例, 探討降級對物種保育可能帶來的影響。這些案例顯示, 降級雖有助於資源的有效分配, 卻也可能引發法規保障弱化、資源轉移, 以及公眾與政策支持減少等風險。高鼻羚羊的降級獲得相對廣泛的支持, 顯示在評估過程中及早且廣泛地納入當地專家、評估人員、資助者與其他利害關係人, 有助於促進溝通與決策的順利推動, 進而降低降級對物種後續復原的影響。此外, IUCN的物種綠色狀態評估(Green Status of Species)作為紅皮書的補充工具, 提供了衡量保育進程的新指標, 有助於決策者確保降級不會削弱長期保育工作的成效。

## ROLE OF THE IUCN RED LIST AND THE IMPLICATIONS OF CATEGORY CHANGES

The International Union for Conservation of Nature (IUCN) Red List of Threatened Species has become the global standard for data on species’ extinction risk and is arguably one of the most important tools used by researchers, conservationists, and governments in conserving and managing threatened species (Betts et al., 2020; Rodrigues et al., 2006). The list provides robust and objective information across all taxa, and IUCN periodically reassesses species to ensure information on the list is up to date.

Although the IUCN Red List emphasizes that assessments alone should not determine conservation priorities, their outcomes strongly influence how conservation resources are actually allocated (Betts et al., 2020); species at greater risk of extinction are generally considered more in need of conservation. This raises important questions about how conservation resource allocation may change following a conservation success and subsequent downlisting, an issue that has received little systematic attention.

Downlisting is an important goal of species conservation worth celebrating because it not only indicates a reduced extinction risk but also allows limited conservation resources to be redirected to species in more urgent need. For instance, after the humpback whale (*Megaptera novaeangliae*) was downlisted from vulnerable (VU) to least concern (LC) on the list in 2008, conservation professionals advocated for adjusting its local conservation status to reflect this success to ensure that limited resources could be redirected to more highly threatened species (Bejder et al., 2016).

However, downlisting sometimes raises concerns that species may receive less attention and support than they require because of how the list is used by conservation decision makers. The case of the woylie (*Bettongia penicillata*), a small marsupial in Australia, is a prime example. After being removed from state, national, and international threatened species lists in 1996 following successful recovery efforts, the species declined rapidly by approximately 75% from 2001 to 2006, leading to its reclassification as endangered (EN) (Groom, 2010). This highlights the risks of prematurely shifting resources away from downlisted species and emphasizes the need for continued monitoring and conservation efforts even after downlisting (Scott et al., 2010).

To reduce the chance of downlisting undermining species recovery, the IUCN follows the 5‐year rule. This rule requires that species considered for downlisting do not meet higher threat category criteria for at least 5 consecutive years before they are downlisted and that the species be uplisted immediately if urgent threats emerge (IUCN Standards & Petitions Committee, 2024). This 5‐year rule increases the chance that observed improvements reflect genuine, sustained recovery rather than temporary fluctuations. Meanwhile, recent red‐list updates have strengthened guidance to ensure assessors systematically consider long‐term threats, such as climate change, species‐specific risks, and population variability for more accurate listing decisions (IUCN Standards & Petitions Committee, 2024). Complementing this, the IUCN uses green status of species (GSS) assessments to examine species recovery and conservation dependence to provide a broader perspective of extinction risk (Grace et al., 2021). Integrating GSS with red‐list evaluations helps ensure that the downlisting process is accurately communicated and interpreted and maintains conservation support.

However, the downlisting of certain high‐profile species can spark controversy, such as the 2024 proposal to downgrade the black‐faced spoonbill (*Platalea minor*) from EN to LC. This case highlights the importance of clear communication and careful interpretation—assessors must ensure that the red list accurately reflects reality, and communicators must work to prevent good news from being misunderstood. There has been little discussion of the broader impacts of species downlisting, and research quantifying changes in resource allocation before and after downlisting remains scarce. This is partly because genuine downlisting events remain relatively rare.

## FREQUENCY OF DOWNLISTING

Over the last few decades, increased data on species’ abundance and distribution have allowed the IUCN to assess more species for extinction risk. Currently, over 45,000 species are listed as threatened—a designation covering critically endangered (CR), EN, and VU categories. These categories represent over one quarter of all assessed species.

**FIGURE 1:**
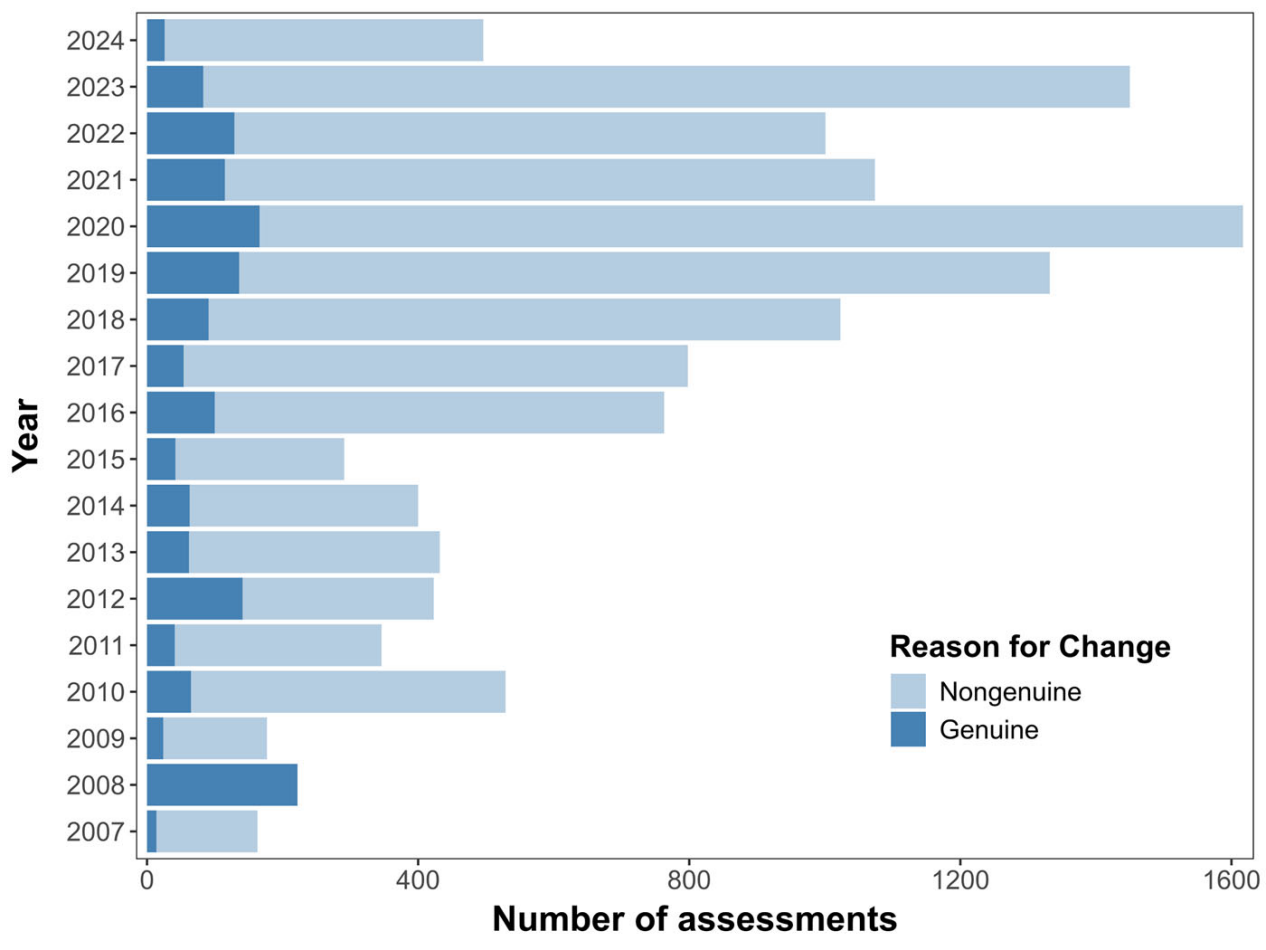
Annual number of International Union for Conservation of Nature (IUCN) Red List category changes (2007–2024) relative to whether the reason for the change was genuine (real population improvement) or not genuine (reasons not related to population improvement). Changes stemming from a listing error are grouped under not genuine. In 2008, only genuine changes were reported by the IUCN.

Despite the increase in assessments, changes in species’ categories are infrequent. From 2007 to 2024, only about 1% of the assessed species changed categories each year; just 5–33% of these annual changes reflected genuine shifts in population status (IUCN, 2024a; Figure 1). Most reasons for category changes were not genuine, for example, they were based on new information or taxonomic rearrangements. This raises an often‐overlooked question: how common is downlisting on the red list due to genuine population recovery and what impact could downlisting have on these species?

**FIGURE 2:**
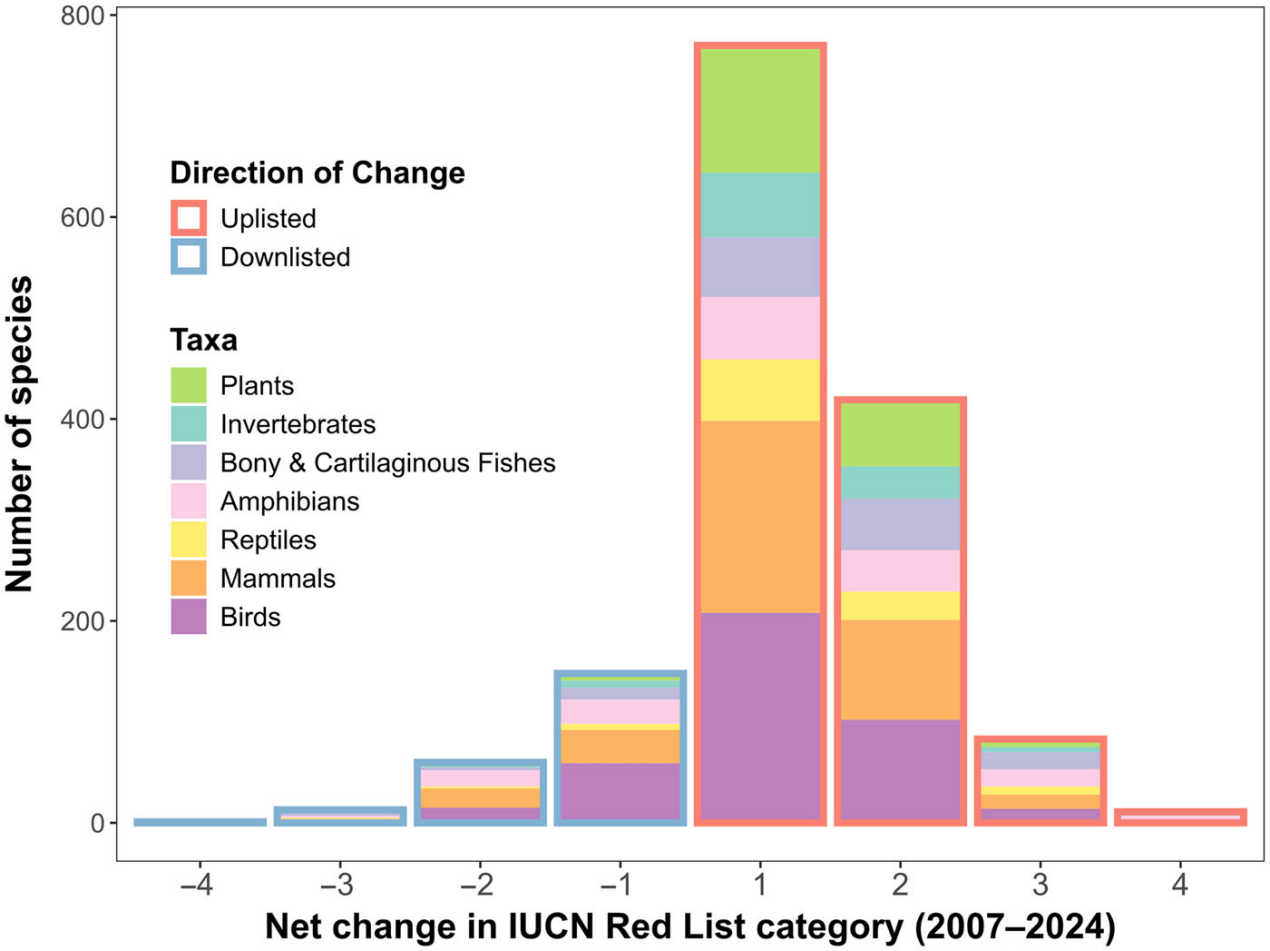
The number of species with a net change in International Union for Conservation of Nature (IUCN) Red List category from 2007 to 2024 due to a genuine change in status (i.e., real population improvement or worsening). Net changes are calculated as the difference in category scores between 2007 and 2024. Categories are scored from highest to lowest extinction risk as follows: 7, extinct; 6, extinct in the wild; 5, critically endangered; 4, endangered; 3, vulnerable; 2, near threatened; and 1, least concern. For example, a net change of +1 indicates uplisting by one category (e.g., from least concern to near threatened), and −1 indicates downlisting by one category (e.g., from endangered to vulnerable). Some historical IUCN subcategories were mapped to the current classification system for consistency: possibly extinct and possibly extinct in the wild were treated as critically endangered; not threatened, low risk, lower risk or conservation dependent, and low risk or near threatened were treated as near threatened; and low risk or least concern was treated as least concern. Changes unrelated to population improvement or worsening (not genuine changes) were excluded, as were 10 species that were data deficient either before or after the changes, 7 species that only changed subcategories within the same main category, and 2 species with no status change despite their appearance in the summary report.

We examined how species’ categories on the IUCN Red List changed from 2007 to 2024. Data were sourced from IUCN summary statistics (IUCN, 2024a) and the R package rredlist (Gearty & Chamberlain, 2022). Of the 163,040 assessed species, 1,511 had at least one genuine status change, leading to 1,505 species with a final change in red‐list category by the end of 2024 (Figure 2). Most cases (85%) involved uplisting to a higher extinction risk, whereas only 15% involved downlisting. Among 222 downlisted species, the majority (66.7%, 148 species) shifted down by one category. Only 0.05% of assessed species were downlisted by 2 or more categories from 2007 to 2024; one species was downlisted by 4 categories.

**FIGURE 3:**
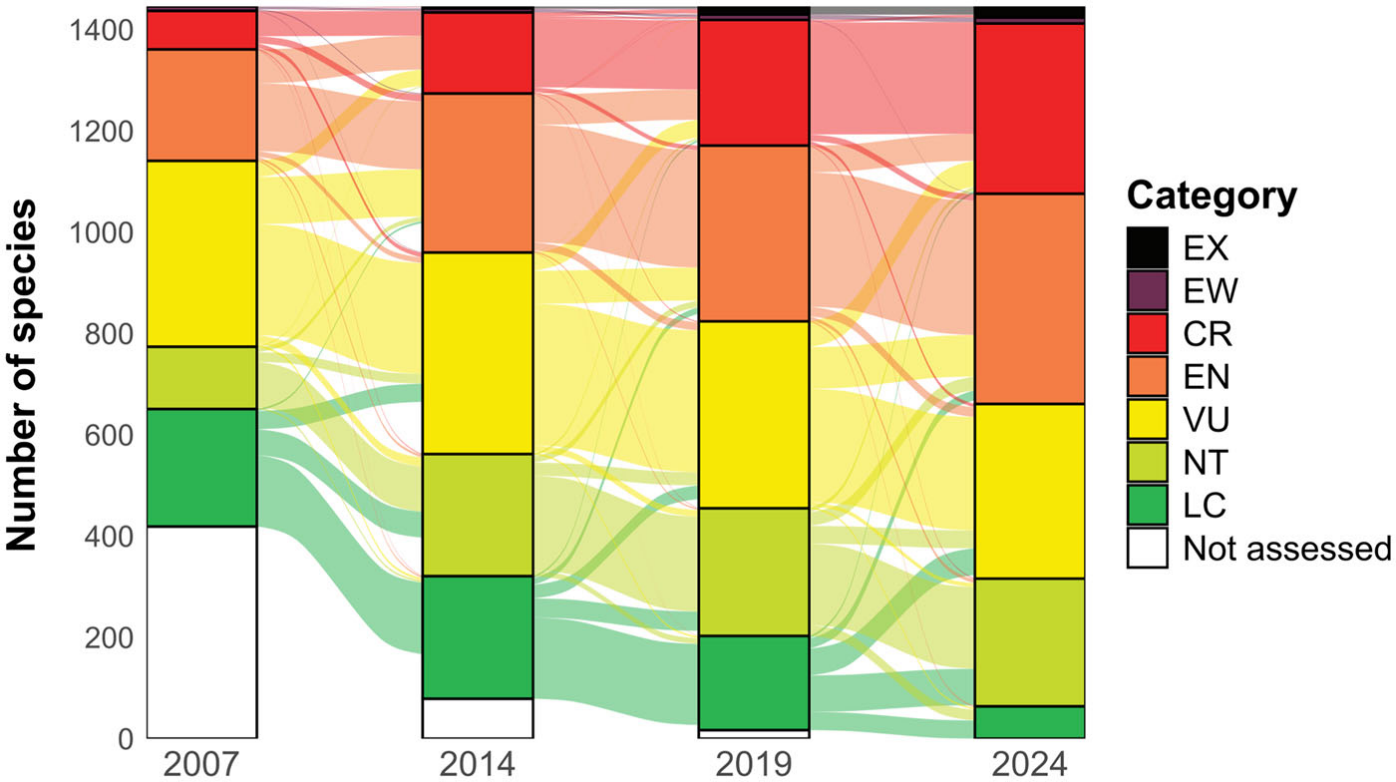
Number of species that had their International Union for Conservation of Nature Red List categories changed in 2007–2014 (7 years), 2014–2019 (5 years), and 2019–2024 (5 years). Category changes are shown for 1,466 species (from a total of 1,511 species that underwent genuine [real population improvement] category changes) assessed from 1988 to 2024 (EX, extinct; EW, extinct in the wild; CR, critically endangered; EN, endangered; VU, vulnerable; NT, near threatened; LC, least concern). The flow shading represents the net transitions between red‐list categories due to genuine changes documented at the start and end of each period, rather than reflecting precise year‐by‐year number of species in the risk categories. Sixty‐five species were excluded due to difficulties in transcribing assessments into the current category system, inconsistent scientific names, or involvement in nongenuine changes between 2 genuine changes.

Over time, more species have been shifted from lower risk to higher risk categories than the reverse. The largest proportion of these changes involved species initially classified as VU moving to EN (Figure 3). Critically endangered species were an exception to this trend. There has been a greater tendency to downlist CR species to categories below EN rather than uplisting them to extinct (EX). This pattern underscores the effectiveness of the red list in alerting the conservation community to take action to prevent CR species from going extinct.

That there were more uplistings than downlistings is concerning because it suggests that more species are being increasingly threatened over time. However, in some cases, conservation efforts have effectively reduced threats, and with growing global attention and increasing investment in biodiversity conservation, more species are expected to recover and be downlisted (Langhammer et al., 2024; Seidl et al., 2020, 2021). This trend represents an ideal outcome and should be celebrated as a substantial conservation achievement. However, the downlisting process has not always been smooth. In some high‐profile cases, it has been accompanied by controversy and disenfranchisement among those instrumental to a species’ recovery, highlighting the need for careful preparation and communication to ensure these positive changes are well understood and supported. Such controversy is particularly notable for flagship species that attract considerable public interest and funding. We examined 4 such examples: saiga antelope (*Saiga tatarica*), giant panda (*Ailuropoda melanoleuca*), red‐crowned crane (*Grus japonensis*), and black‐faced spoonbill.

## RESPONSES TO THE DOWNLISTING OF FLAGSHIP SPECIES

The saiga antelope, giant panda, red‐crowned crane, and black‐faced spoonbill are all charismatic animals that play an important role as flagship species in their respective ecosystems in Asia. They also act as umbrella species in that their protection likely benefits many other species that live in sympatry (Choi et al., 2022; Li & Pimm, 2016; Milner‐Gulland & Singh, 2016; Nakamura, 2018). All 4 species underwent rapid population decline but are now showing signs of recovery, demonstrably aided by conservation interventions. Local, national, and international stakeholders initiated research programs, started long‐term monitoring, implemented habitat protection, and banned poaching soon after these species were listed as at extreme extinction risk (Cano‐Alonso et al., 2023; Milner‐Gulland & Singh, 2016; Momose et al., 2019; Wei et al., 2018).

The saiga antelope represents a downlisting case widely recognized as a conservation success. Once on the brink of extinction due to poaching, habitat loss, and disease, the species has recovered rapidly over the past 2 decades, largely due to intensive conservation interventions led by the Kazakh government (IUCN SSC Antelope Specialist Group, 2023). As a result, it was downlisted from CR to NT in 2023. The species’ recovery had been well documented, and the conservation community was prepared for its eventual ineligibility for higher threat categories. Moreover, stakeholders were actively engaged in the reassessment to ensure a smooth, uncontroversial process. The downlisting was communicated as a conservation success, reinforcing the importance of effectively informing and involving stakeholders in status changes to maintain transparency and support for ongoing conservation efforts.

Giant panda conservation is widely celebrated as a success, although not without some controversy (Mallon & Jackson, 2017). It was listed as EN in 1990 and 2008 but was subsequently downlisted to VU in 2016, following conservation efforts, including habitat protection, antipoaching enforcement, and large‐scale forest restoration (Swaisgood et al., 2016). These actions were further supported by costlier ex situ measures, such as captive breeding and reintroduction programs. Critics argue that downlisting could reduce conservation funding and efforts, potentially undermining ongoing efforts needed to support its population (Wei et al., 2018).

Two cases involving migratory bird species presented greater challenges. The red‐crowned crane's downlisting from EN to VU in 2021 sparked controversy. This assessment was based on the slowing of the global population decline, largely due to the growth of the nonmigratory Japanese population (BirdLife International, 2021). Some argue that downlisting may create a misleading impression that the species as a whole is no longer at risk (CBCGDF, 2020; Ono & Matsuo, 2020). Indeed, the Chinese population declined by over 90% in recent decades and continues to face threats, and the growing Japanese population relies heavily on conservation interventions, such as supplementary feeding (BirdLife International, 2021). There is concern that downlisting could reduce the resources available for conservation, worsening the species’ overall situation (CBCGDF, 2020; Ono & Matsuo, 2020).

In the most recent case, the black‐faced spoonbill, listed as CR in 1994 and downlisted to EN in 2000, is being proposed for further downlisting to LC (BirdLife International, 2017; BirdLife International Forums, 2024). The species has benefited from long‐term, coordinated conservation efforts across multiple countries (habitat protection and management, disturbance removal, and nest site enhancement) that played a key role in its recovery (Cano‐Alonso et al., 2023; Kwon, 2017; Lin et al., 2024; Sung et al., 2018). Although the population has steadily increased, this downlisting proposal has sparked intense debate. Critics argue that the species’ threatened status was instrumental in enabling critical conservation actions, such as the establishment of protected areas and the cessation of military activities on key breeding grounds (Kwon, 2017; NIE, 2024). Furthermore, assigning LC status could lead to significant underestimation of the ongoing threats the species continues to face. As the population increases, individuals have become increasingly concentrated in limited habitats and there has been little expansion of breeding or wintering areas. This makes them especially vulnerable to threats, such as climate change, avian influenza, and the development of green energy infrastructure, including solar panels and wind farms (Lai et al., 2025; Pickett et al., 2018). These complexities underscore the importance of comprehensive consultation and strong local stakeholder engagement to ensure that downlisting decisions are well supported and that broader impacts on conservation funding, policy, and management are considered carefully.

Public responses to downlisting vary significantly among species. Although concerns about reduced conservation resources arose for the giant panda, red‐crowned crane, and black‐faced spoonbill, the saiga antelope's downlisting was comparatively smooth, despite its being conservation dependent (Milner‐Gulland & Mallon, 2024). Its broader acceptance may have been due to more comprehensive and timely inclusion of local experts and stakeholders that fostered greater consensus within the conservation community on both the assessment process and its results.

## EFFECT OF DOWNLISTING ON A SPECIES

### National conservation priority

The national protection status of a species in many countries is informed by the IUCN Red List alongside consideration of national concerns and priorities. The red list is an index of extinction risk and does not inherently connote conservation priority or carry direct legal ramifications in most countries. For example, the giant panda is still listed as a class I protected species, the highest protection category in the revised National Key Protected Wild Animal List (NKPWA) of China (NFGA, 2021), despite its IUCN downlisting in 2016 (Swaisgood et al., 2016). In some countries, such as Korea and China, relevant policies directly refer to the IUCN Red List category when determining national protection status (Jiang et al., 2016), which is contrary to IUCN guidance on appropriate use of red‐list data (IUCN, 2022). Although changes in red‐list status should not dictate national conservation policy, they may still influence decision‐making, underscoring the need for policy makers to apply red‐list information appropriately.

### Diminished legal foundation for a downlisted species’ protection

Downlisting of a species may lead to similar action at the national level, leaving prosecutors, conservation practitioners, and the public with a weaker legal basis for protection of a species in some countries. Although a relaxation of protection mechanisms might appropriately reflect the lower extinction risk of a downlisted species, it is possible that threats could escalate rapidly. For example, in China, it is forbidden under the *Conservation of Wild Animals Act* to hunt, capture, kill, sell, acquire, or use any species on the NKPWA. Violations can lead to criminal liability and penalties, the severity of which depends partly on the protection level of species. If a species is removed from the NKPWA in response to downlisting, it is possible that threats could rapidly increase.

### Potential shifts in conservation and research resourcing

A major concern in debates over species downlisting is the potential for reductions in conservation and research funding (Ma et al., 2018; Mallon & Jackson, 2017; Swaisgood et al., 2018). Some conservation funding programs prioritize species with higher red‐list categories, for example, the Conservation Leadership Programme (2024) (DD, VU, EN, and CR prioritized), the Mohamed bin Zayed Species Conservation Fund (2024) (EN, CR), the Ocean Park Conservation Fund (2024) (CR, EN, VU, NT, DD), and the Zhilan Foundation in China (2024) (CR, EN, VU, DD). A review of conservation funding for threatened birds in Australia from 1993 to 2000 shows that most resources are directed toward taxa closest to extinction (Garnett et al., 2003). This has raised concerns that downlisted species may face funding cuts, despite many of them being requiring continued management to maintain their populations.

However, funding patterns may not always align with threat levels. Hermoso et al. (2017) found that from 1992 to 2013, the European Union's key conservation funding program, LIFE‐Nature, disproportionately allocated funds to LC species in total budget share and in per‐species funding, indicating potential overfunding of LC species compared with more threatened ones. Similarly, Guénard et al. (2025) analyzed more than 14,600 conservation funding projects worldwide over 25 years and found that although less than one quarter of the species covered by these projects are classified as nonthreatened (NT and LC), they still receive more than one third of the total funding.

Funding decisions and conservation priorities are shaped by factors other than extinction risk, such as species charisma and public interest, which can outweigh the influence of red‐list status (Colléony et al., 2017) and lead to continued funding of charismatic and well‐known species after downlisting. However, for species lacking public appeal, losing threatened status might make securing resources even harder, reducing the chance of cementing recovery gains. There is limited empirical evidence on how funding changes are associated with a species’ red‐list status revision. More research is needed to understand these dynamics and assess whether downlisting genuinely affects conservation resourcing and whether that impact may actually be desired and appropriate given the reduced extinction risk.

### Potential reductions in conservation efforts at the community and ecosystem levels

Downlisting a species could have implications beyond the focal species itself; conservation efforts at the community and ecosystem levels may be affected. Many species are ecological flagships and thus act as catalysts for habitat protection, research, and policy decisions that benefit a wide range of sympatric species. When a species that has played a key role in these processes is downlisted, it may subtly shift conservation priorities and make it more challenging to justify new protections or maintain existing conservation efforts at the same scale. For example, species recognized as conservation priorities can help drive the establishment of protected areas. In East Asia, waterbirds, such as the black‐faced spoonbill, have contributed significantly to wetland conservation efforts (Choi et al., 2022). Their presence at a site has sometimes qualified the site for international recognition, such as Ramsar designation. Although downlisting does not directly lead to the removal of existing protected areas, it may reduce the perceived urgency for new designations, particularly where economic and political pressures already constrain conservation (Symes et al., 2016).

Beyond habitat protection, species at high risk of extinction can serve as focal points for environmental impact assessments and mitigation measures in development projects. In some cases, detailed movement and ecological data from threatened species have played a crucial role in assessments of the impacts of renewable energy infrastructure, urban expansion, and coastal development on wildlife (Lai et al., 2025). When a species is downlisted, it may no longer trigger the same level of scrutiny in development planning, potentially reducing compensatory measures, such as habitat offsets. However, the extent to which this occurs may depend on government policies and conservation commitments. For example, despite downlisting of the giant panda, its conservation remains a national priority in China, ensuring continued investment and habitat protection (SFA, 2015).

We do not suggest that these shifting dynamics imply that species should remain in an inflated (and incorrect) extinction risk category for the sake of maintaining conservation effort. Rather, we believe they highlight the importance of anticipating and addressing the broader ecological and policy implications of species downlisting, ideally well ahead of the downlisting event occurring. Understanding these effects can help stakeholders develop proactive strategies to ensure that conservation momentum is maintained, even as species recover and move out of higher risk categories.

### Shifts in symbolism of flagship species

Following downlisting, public interest in the conservation of the downlisted species could decrease, leading to increased difficulty in recruiting volunteers and observers to continue needed conservation activities and monitoring work. For some flagship species, this may weaken mobilization of support, particularly if conservation messaging has emphasized rarity as the primary reason for concern. Such framing can inadvertently imply that recovery diminishes the need for continued conservation efforts.

In response, some may propose identifying a new flagship species to continue the conservation work at the community or ecosystem level, though this can be time‐consuming and resource intensive. These dynamics underscore the importance of providing substantial notice ahead of potential downlisting so that practitioners can anticipate changes and adapt. Diversifying conservation narratives—beyond extinction risk to include ecological roles, cultural value, or indicator function—can also help sustain support as species recover.

## DOWNLISTING WITHOUT COMPROMISING RECOVERY OF THREATENED SPECIES

### Ensure sustainable conservation commitments

When planning conservation strategies, it is crucial to prioritize systematic, long‐term approaches that are sustainable. A key concern is that shifts in donor priorities following a downlisting may lead to population collapses in conservation‐dependent species if human interventions are suddenly withdrawn. Therefore, for conservation practitioners, NGOs, and policy makers, it is essential to recognize that interventions, such as supplementary feeding or captive breeding programs, could quickly increase the population size of a threatened species. However, if habitat degradation and loss persist, these efforts may create populations that exceed the reduced carrying capacity of their natural habitats, making them practically and financially unsustainable in the long run.

To avoid this, conservation funding must be structured for long‐term sustainability rather than short‐term population gains. Donors, funding agencies, and governments play a crucial role in prioritizing habitat restoration, ecosystem resilience, and self‐sustaining populations over intensive efforts focused on a single species—particularly charismatic species that attract widespread public attention. Aligning funding models with sustainable, nature‐based conservation strategies will help maintain stability in conservation efforts as species transition out of higher risk categories.

### Clarifying downlisting through IUCN Red List and Green Status integration

In its red‐list assessments, the IUCN follows science‐based guidelines that may not always align with public perceptions of a species’ conservation status. The extent to which a species depends on ongoing conservation efforts or what the consequences of withdrawing those efforts would be is not explicitly indicated. When the IUCN Red List Categories and Criteria were first published in 1994, a conservation‐dependent (CD) subcategory was included under the lower risk (LR) category to recognize that some species require continued conservation action to remain stable (IUCN, 1994). However, this subcategory has now been removed to reflect the fact that conservation dependence can occur across all threat categories (IUCN, 2001a, 2001b; IUCN Standards & Petitions Committee, 2024). Regardless of the existence or otherwise of explicit recognition of conservation dependence in the red‐list criteria, downlisting can sometimes be misunderstood because it does not necessarily indicate a reduced need for conservation action.

The GSS assessment partially addresses this issue because the progress of a species’ recovery and the impact of conservation actions are evaluated (Akçakaya et al., 2018; IUCN, 2021). Unlike the red list, GSS assessments are based on a species’ recovery stage and distinguish between species that are ecologically restored and those that, despite improved population numbers, remain depleted or conservation dependent. In a GSS assessment, a “species recovery score,” ranging from 0% to 100% (100% is full recovery), is assigned. For instance, the black‐faced spoonbill's GSS assessment resulted in a recovery score of 35%, which means it was classified as “largely depleted” and having a high “conservation legacy” and high “conservation dependence” (Cano‐Alonso et al., 2023). This case underscores the importance of integrating GSS assessments with red‐list assessments and of ensuring this integration is recognized in regulatory instruments and conservation funding mechanisms. Although the black‐faced spoonbill may eventually qualify for downlisting on the red list, its GSS assessment ensures that its continued need for conservation is not forgotten.

A GSS assessment became an optional component of red‐list assessments in 2020 (IUCN, 2024b), and its use can help mitigate concerns over downlisting by providing a clearer picture of long‐term conservation needs. A species’ red‐list status may improve, but if its GSS assessment indicates continued reliance on conservation, downlisting should not inadvertently undermine funding, policy action, or habitat protections essential to sustained recovery.

However, the GSS does not have the same level of recognition or use as red list, and most stakeholders still primarily rely on the red list for conservation decisions. Therefore, it is crucial to promote greater awareness and integration of the GSS with red‐list information to foster a more accurate and constructive interpretation of downlisting. Researchers and conservation practitioners should emphasize that rather than full recovery, downlisting reflects the *process* of stabilization or recovery (Swaisgood et al., 2018). Downlisting should be recognized as a conservation goal and achievement, rather than an indication that conservation actions are no longer needed (Mallon & Jackson, 2017).

### Ensuring transparent communication and a smoother downlisting process

Although red‐list assessments strive to be objective, stable, and neutral, recent controversies surrounding downlistings underscore the need for clear communication with stakeholders, especially local experts, to ensure a shared understanding of the process. Many concerns around downlisting fall outside IUCN's scope or have already been considered in red‐list assessments, making transparent communication essential to maintaining public confidence in the system.

Despite broad agreement that downlisting is the ultimate goal, some conservation practitioners perceive certain downlistings as too fast. For example, the proposal to reassess the black‐faced spoonbill from EN to LC raised concerns, despite its population recovery being well documented. In reality, such cases often reflect delayed reassessments rather than genuinely abrupt status changes. Due to capacity constraints, species reassessments often occur years apart—sometimes over a decade (Rondinini et al., 2014). As a result, some species may continue to improve and surpass multiple category thresholds before their next assessment. In this case, the problem is not that species are being downlisted too quickly, but rather that some species are not reassessed sooner despite no longer meeting higher threat criteria. Although the IUCN's 5‐year rule requires that species meet criteria for a lower risk category for at least 5 years prior to downlisting, this condition is only evaluated at the time of reassessment and may already be satisfied if recovery occurred earlier. To support transparency and planning, species showing clear signs of recovery could be more prominently flagged in red‐list documentation. This would allow conservationists, policy makers, and donors to better anticipate future changes and ensure smoother resource allocation and conservation planning.

We hope our ideas will help shape the development of appropriate, ongoing conservation strategies for downlisted species by promoting early preparation, open communication among stakeholders, more prominent application of the GSS assessment, and equipping of the conservation community to better deal with the consequences of its own successes.
